# Complications, Urinary Continence, and Oncologic Outcomes of Laparoscopic Radical Prostatectomy: Single-Surgeon Experience for the First 100 Cases

**DOI:** 10.1155/2011/606505

**Published:** 2011-07-14

**Authors:** Takashi Imamoto, Yusuke Goto, Takanobu Utsumi, Miki Fuse, Koji Kawamura, Naoto Kamiya, Yukio Naya, Hiroyoshi Suzuki, Yukihiro Kondo, Tomohiko Ichikawa

**Affiliations:** ^1^Department of Urology, Graduate School of Medicine, Chiba University, Chiba 260-8670, Japan; ^2^Department of Urology, Toho University Sakura Medical Center, Sakura 285-8741, Japan; ^3^Department of Urology, Teikyo University Chiba Medical Center, Ichihara 299-0111, Japan; ^4^Department of Urology, Nippon Medical School, Bunkyo-ku, Tokyo 113-8602, Japan

## Abstract

*Objective*. The aim of the present study was to evaluate initial learning curves of laparoscopic radical prostatectomy (LRP) with regard to complications, urinary continence, and oncologic outcome. *Materials and Methods*. We retrospectively reviewed 100 consecutive patients with clinically localized prostate cancer. All 100 patients underwent LRP performed by the same urologist at one institution. *Results*: Mean operating time (208.4 ± 48.6 min), estimated blood loss (495.8 ± 436.5 mL), allogeneic blood transfusion rate (0%), and intraoperative complications diminished with surgical experience. Positive margin rate varied greatly among pathological stage (positive margin rates: pT2 = 20.5%; pT3 = 63.0%). A trend towards reduction of positive surgical margins in pT2 cases was apparent with increasing experience. Intraoperative and early complications occurred in 2.0% of patients. In all patients, 85.9% used none or no more than one pad per 24 h at 6 months postoperatively. Prostate-specific antigen recurrence was seen in only 2 patients. *Conclusions*. In the present series of 100 patients, our retrospective evaluation confirms that LRP provides satisfactory results.

## 1. Introduction

The development of minimally invasive surgical techniques has resulted in a greater focus on achieving optimal functional outcomes in patients undergoing radical prostatectomy. Laparoscopic radical prostatectomy (LRP) is currently performed mainly in France [1, 2]. Surgeons with experience in this field have described various advantages of the laparoscopic procedure versus the open approach, such as optical magnification, less blood loss, less postoperative pain, and more rapid resumption of normal activities [3–5].

Since 2007, LRP has been the modality of choice for localized prostate cancer at Chiba University Hospital. One reason for starting with radical prostatectomy was that although this approach is technically more difficult than laparoscopic nephrectomy or adrenalectomy, it is also less dangerous and has fewer life-threatening potential complications. Initially, one surgeon from our department started the program with transperitoneal LRP after intensive training for 10 months in a reference center with a great deal of experience in LRP [6]. 

Laparoscopic prostatectomy is declining as a procedure for prostate cancer around the world. In fact, in the United States, this represents only 5% or less of the procedures done for prostate cancer. Robotic prostatectomy is currently the most widely done procedure in the US, At present in Japan, robotic prostatectomy has not yet been widely done because of some problems around the medical economy. To meet institutional standards for laparoscopic radical prostatectomy, a surgeon must have performed more than 10 procedures under the guidance of experts in Japan. Because it can be rather difficult to introduce laparoscopic prostatectomy to an institution that is unfamiliar with the procedure, it is necessary to use a stepwise program to teach laparoscopic surgical techniques, to reduce the duration and morbidity of learning curves. The goal of this study was to evaluate the first 100 patients treated by LRP. We focused on intraoperative early and late complications, positive surgical margins, urinary continence, and prostate-specific antigen (PSA) recurrence.

## 2. Patients and Methods

Between April 2007 and May 2010, a total of 100 consecutive patients underwent LRP for localized prostate cancer performed by the same surgeon at one institution (Department of Urology, Chiba University Hospital, Chiba, Japan). Patients were decodified and ordered chronologically from number 1 to number 100 for this paper. The indication for lymphadenectomy was a >3% probability of lymph node metastasis according to the Kattan nomogram [7].

Overall preoperative data are shown in Table 1. In 1 patient, surgery was reconverted to open surgery for rectal injury repair. Mean patient age was 65.2 ± 4.9 years (range, 50–73 years). Some of the 100 patients were initially diagnosed with localized prostate cancer at another hospital then referred to our institution for LRP. Four of those patients had received neoadjuvant hormonal therapy at the other hospital. Mean body mass index (BMI) was 23.3 kg/m^2^ (range, 18.1–27.9 kg/m^2^). Of the 100 patients, 51 had undergone abdominal operations before LRP. The most frequent operations before LRP were appendectomy (36%), gastrectomy (6%), herniotomy (3%), colorectal surgery (3%), and cholecystectomy (2%).

The single surgeon was assisted by an experienced surgeon during the first several cases and by the newer generation of surgeons thereafter. The latter were helped by residents as the scope holder. LRP was initially performed using the transperitoneal approach according to the methods of Guillonneau and Vallancien [1]. This procedure was modified to use an entirely extraperitoneal approach after Patient 8. Patients 34–100 were operated on using the same technique, but with a modified apical dissection to diminish the positive margin rate at the apex. This technique involves dissecting the neurovascular bundle off the apex before transecting the urethra. Urethrovesical anastomosis was initially performed with interrupted stitches but was modified to use a running suture after Patient 16. The procedure was modified to include restoration of the posterior aspect of the rhabdosphincter (RS) after Patient 65. Prior to completion of the vesicourethral anastomosis, the posterior fibrous tissues of the sphincter were joined to the residual Denonvilliers' fascia on the posterior bladder wall 1-2 cm cephalad and dorsal to the new bladder neck [8, 9].

Side-specific intrafascial dissection of the neurovascular bundle was usually performed in prostate sides with no palpable nodules, biopsy Gleason score 3 + 3 or 3 + 4, maximum percentage of positive biopsy <10% (depending on the location of the biopsy), particularly when positive cores were located medially, or in the absence of suspected extracapsular extension (ECE) on magnetic resonance imaging (MRI). However, a prostate side with a single positive biopsy with Gleason score 4 + 3 or a prostate side with a maximum percentage of cancer >10% compromising a medial core without signs of ECE may be suitable for intrafascial dissection. These criteria should not be taken as a strict rule but rather as a general guideline [10]. These specific indications for nerve-sparing LRP limited the number of patients who underwent the procedure (*n *= 24, 18 unilateral and 6 bilateral). Sufficient erectile function for sexual intercourse was assessed between patients who underwent uni- and bilateral nerve sparing, respectively.

We reviewed the following data: patient age and BMI; operating time (OR time); anastomosis time; estimated blood loss; hospital stay; presence or absence of urinary leak; duration of postoperative bladder catheterization; intraoperative complications; immediate postoperative complications (appearing within the first month after surgery); long-term complications (appearing after the first postoperative month); TNM staging (Union Internationale Contre le Cancer 2002 classification); surgical margin status. Biochemical recurrence of prostate cancer was defined as two consecutive increases in serum PSA level by >0.1 ng/mL. To study the learning curve, all variables were calculated for every 25 consecutive patients operated on by the surgeon. Statistical significance was assessed using Student's *t*-test and the log-rank test. Probability values of *P* < 0.05 were considered to indicate significance.

## 3. Results

**Figure 1:**
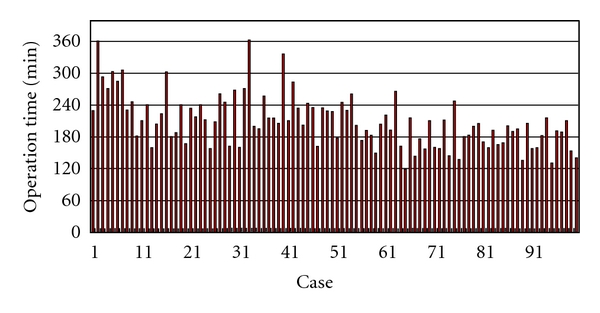
Operation time for all patients.

**Figure 2:**
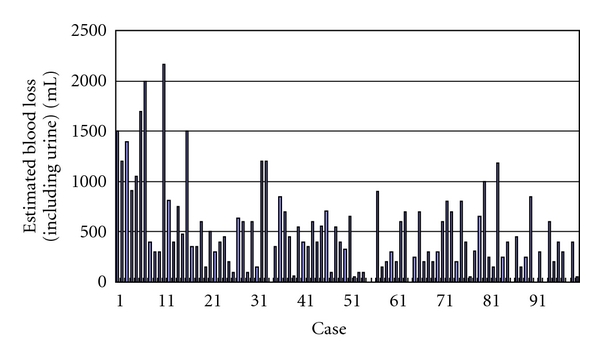
Estimated blood loss (including urine) for all patients.

Median duration of followup was 25.0 months (range, 10.2–47.7 months). Mean mass of removed prostate was 43 g (range, 16–122 g). Mean OR time was 208 min (range, 119–362 min) (Figure 1). OR time tended to decrease over time with surgical experience, with approximately 40–50 cases required for the surgeon to reach a stable OR time. Mean estimated blood loss was 496 mL (range, 10–2160 mL) (Figure 2). Eleven patients showed blood loss >1000 mL, but hemoglobin levels immediately after surgery were >10 g/dL in all patients. This suggests that a considerable amount of urine was included in the estimated intraoperative blood loss, suggesting that the true amount of blood loss was even less than the present data indicate. Indeed, no patients required allogeneic blood transfusion. Estimated blood loss tended to decrease over time. In almost all patients, the drain was removed during the first 4 days postoperatively. The criteria for drain removal were a daily volume drained <50 mL and no suspicion of urinary leakage. 

Median duration of catheterization was 6.6 days (range, 6–18 days). Cystourethrographies were performed in 58 consecutive patients on postoperative day 6 or 7. Most patients showed no leakage or only a small leak of contrast medium at the anastomotic site. After these results, we decided to leave the catheter for 6 days in all subsequent patients and perform withdrawal without cystourethrography unless the patient showed persistent urine leakage through the drainage during the postoperative period. In all of the latter cases, the catheter was safely removed 6 or 7 days postoperatively without high risk of incontinence, stricture, or leakage. Only 2 patients experienced vesicourethral anastomotic stricture.

**Figure 3:**
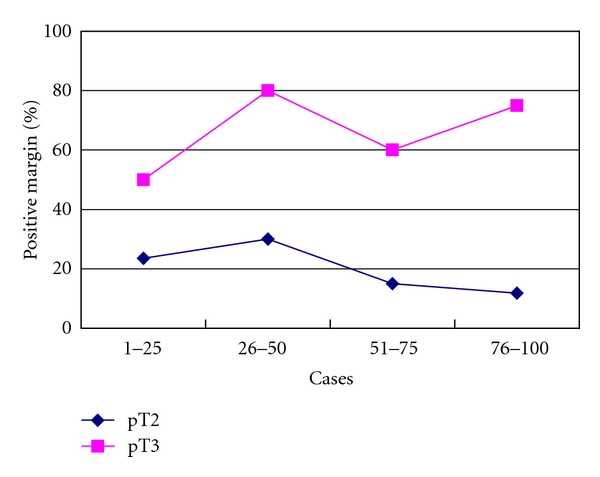
Percentage of positive surgical margins according to pT category and number of cases operated on consecutively.

**Figure 4:**
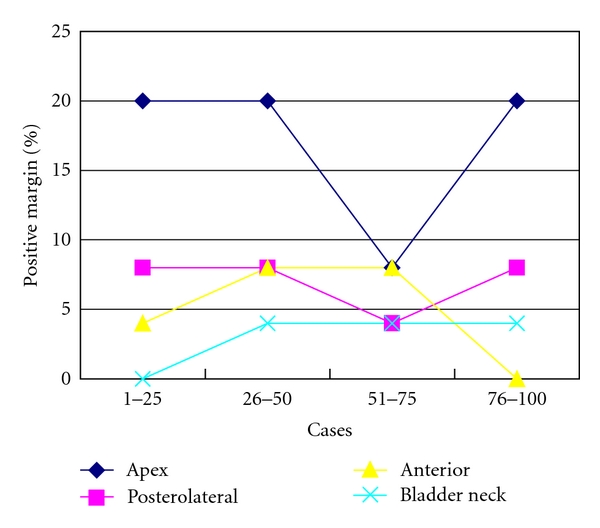
Percentage of positive surgical margins according to location and number of cases operated on consecutively. The technique of apical dissection was modified after Patient 33.

**Figure 5:**
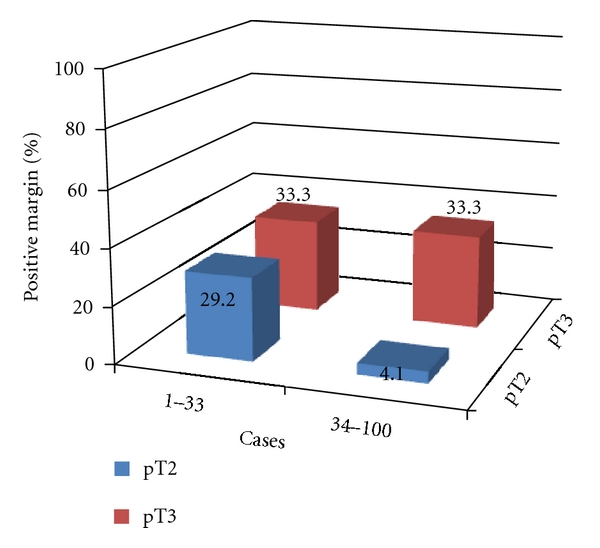
Percentage of positive surgical margins according to pT and number of cases operated on consecutively. The technique of apical dissection was modified after Patient 33.

Pathological stages were as follows: pT0, 1 patient; pT2a, 8 patients; pT2b, 64 patients; pT3a, 24 patients; pT3b, 3 patients (Table 2). In 32 cases, surgical margins were positive. A reduction in positive margin rate was seen over time for pT2 cases (Figure 3). Positive margins in pT3 cases did not change significantly with surgeon experience. Location of margins and changes with the number of cases operated on are shown in Figure 4. Apical and posterolateral margins were the most frequent. The surgical technique changed over time. During the first 33 cases, the positive margin rate at the apex was 29.2% (7 of 24) for pT2 and 33.3% (3 of 9) for pT3 cases. Patients 34–100 received a modified apical dissection: instead of cutting the urethra before dissecting the neurovascular bundles off the prostate, transection was performed only once the neurovascular bundles had been completely separated from the prostatic apex. Margins at the apex with this modified dissection fell to 4.1% (2 of 49) in pT2 and 33.3% (6 of 18) in pT3 cases (Figure 5). Lymphadenectomy was performed in 13 patients, all of whom showed tumor-free lymph nodes. PSA recurrence was seen in only 2 patients. 

Intra- and immediately postoperative complications occurred in 2 of 100 patients (2.0%) and are listed in Table 3. Major intraoperative and immediately and long-term postoperative complications except for inguinal hernia were comparatively rare throughout this series and did not change significantly with surgeon experience. An intraoperatively identified rectal injury (under laparoscopic conditions) was immediately sutured after open conversion. Mean duration of postoperative hospitalization was 13 days (range, 8–22 days) and did not change with increasing experience. 

Urinary continence was assessed in all patients. To avoid misinterpretation of assessment and comparability, only the number of pads per 24 h is documented. Of the 100 patients treated with LRP, 55.6% used a maximum of 1 pad per 24 h at 3 months postoperatively, compared to 85.9% at 6 months postoperatively. Sufficient erectile function for sexual intercourse with or without augmentation of erectile function using phosphodiesterase (PDE)5 inhibitors was reported as present in 50.0% and 83.3% of patients who underwent uni- and bilateral nerve sparing, respectively.

## 4. Discussion

Several aspects of LRP are unfamiliar to surgeons who have performed only open retropubic radical prostatectomy. Introducing laparoscopic prostatectomy to an institution that is unfamiliar with the procedure can thus be difficult. Our previous report indicated that intensive training at an experienced institution could improve the learning curve for introduction of LRP to an institution that is unfamiliar with the procedure and could thus prove clinically very useful [6]. That protocol did not cause prolongation of the postoperative course and consequently did not compromise the learning curve or quality of surgical results. 

A comparative study between laparoscopic early and late groups and an open radical prostatectomy group has been reported by Rassweiler et al. [11]. Mean OR time was 218 min for late laparoscopic surgery and 196 min for open surgery. Transfusion rates were 9.6% and 55.7%, respectively. Complications included rectal injuries (1.4% versus 1.8%), lymphoceles (0% versus 6.9%), and anastomotic strictures (4.1% versus 15.9%, resp.). 

Our procedure was initially performed using the transperitoneal approach but has been modified to use an entirely extraperitoneal approach after Patient 8. Guillonneau et al. reported the experience and perioperative complications of LRP after 3 years of experience in a series of 567 patients [12]. The conversion rate to conventional retropubic radical prostatectomy was 1.2%, and the reoperation rate due to major complications was 2.3%. The majority of complications were intraperitoneal: 1 case of sigmoid injury; 2 cases of ileal injury; 6 cases of ileus (1 case needing reintervention); 5 cases of hemoperitoneum; 2 cases of uroperitoneum requiring percutaneous drainage. 

We ligated the dorsal vascular complex (DVC) using a “figure 8” stitch. Currently, we place this stitch immediately before starting dissection of the apex. This approach is of particular interest in the presence of an accessory pudendal artery or whenever an anterior tumor is suspected based on preoperative studies (biopsy or MRI or both). When anterior tumor is suspected, the fat of the anterior surface of the prostate should be removed en bloc with the specimen to minimize the risk of anterior positive surgical margins. The urethra was originally incised before freeing the apices from the NVBs. Today, once the NVBs have been dissected from the apices, the distal attachments of Denonvilliers' fascia and the ischioprostatic ligaments are incised on both sides. The prostate is thus left hanging from the urethra, which is subsequently incised using cold scissors. In agreement with other authors [13], we observed that sectioning the urethra after dissection of the apex decreases the incidence of positive apical margins. The overall rate of positive surgical margin rates after open radical prostatectomy in the series by Wieder and Soloway was reported as 28% [14]. In our study, positive surgical margins were found in 20.5% (15/73) of patients with pT2-tumor and 63.0% (17/27) of patients with pT3-tumor. The oncological evaluation of 1000 LRPs at Montsouris Institute showed results ranging between 6.9% for pT2a and 34% for pT3b tumors [15]. 

Among early cases in the present series, anastomosis was initially performed using interrupted stitches. As the skill of the initially inexperienced surgeon improved, the procedure was modified to use a running suture. This modification reduced the OR time, particularly for later cases. The Foley catheter could be removed earlier throughout this series because of the watertight sutures. After 3 months, 55.6% of patients required 0-1 pad per day. The 6-month follow-up figure was 85.9%. Guillonneau et al. [16] and Türk et al. [17] reported complete continence rates of 76% and 86%, respectively, at the 6-month followup. After a 6-month followup, 85% (*n *= 969) of the 1,300 patients were completely continent, 11.9% (*n *= 136) showed minimal stress incontinence, and 3.1% (*n *= 35) needed >2 pads daily. After 12 months, 91.9%, 6.9%, and 1.2% of patients were completely continent, needed 1-2 pads per day or >2 pads per day, respectively. 

One possible reason for this urinary incontinence is a postoperative deficiency of the RS. Reconstruction of the posterior aspects of the RS has recently been demonstrated to allow rapid recovery of continence after radical retropubic prostatectomy [8]. The previous study evaluated the application of this technique in video-LRP, assessing the percentage of continent patients at 3, 30, and 90 days after catheter removal [9]. This demonstrated that posterior reconstruction of the RS appeared to offer an easy and feasible technique even in a laparoscopic setting, with a significantly reduced time to continence recovery. The authors of the studies previously mentioned concluded that the technical modifications applied achieved a substantial and significant reduction in time to continence with no adverse effects. We also modified our procedure with restoration of the posterior aspect of the RS after Patient 65. Despite the relatively small cohort size, our retrospective evaluation confirmed that this modification tends to provide satisfactory results on early continence. Other current reports suggest the usefulness of posterior RS reconstruction and periurethral suspension stitches during robot-assisted LRP [18, 19].

In the nerve-sparing group, potency rates following uni- and bilateral nerve sparing were 50.0% and 83.3%, respectively. Potency results for uni- and bilateral nerve-sparing open retropubic radical prostatectomy reportedly differ considerably between various centers, reaching up to 86% at 12 months in cases of younger patients with bilateral nerve-sparing and use of PDE5 inhibitors [20–22]. According to Anastasiadis et al., potency rates after LRP (*n *= 230) and open retropubic prostatectomy (*n *= 70) at 1 year after surgery were 30% and 41%, respectively. After preservation of one or both neurovascular bundles, potency rates increased from 37% to 44% for the retropubic approach and from 46% to 53% for LRP. 

Of note in our series was the fact that a relatively low number of patients underwent nerve-sparing procedures. Except for the selection indications, another reason for exclusion from nerve-sparing procedures was the postoperative pathological stage in our series, with pT3 in 27 patients (27.0%). In most published series for radical prostatectomy, the proportion of patients with pT3 tumor has been under 25% [15, 23] suggesting that individual patterns of patient selection as well as PSA screening policies have been employed. Despite this relatively high rate of pT3 surgical pathology, our functional results are equal to the results published for open prostatectomy and LRP [16, 20, 22]. In addition, the number of nerve-sparing procedures is due also to the time required to develop and master the technique to such a proficient level. Stolzenburg et al. [24] reported that outcomes were clearly in favor of the intrafascial technique when evaluating potency results according to patient age, particularly in younger patients (i.e., <50 years: 79.4% and 91.6% for standard and intrafascial procedures, resp.).

A recent study [25] found that postoperative complications, prolonged hospital stay, and late removal of the drainage tube were significantly more frequent in surgeons who just performed LRP procedures occasionally. In the present series, the rate of postoperative positive surgical margins was 20.5% for pT2 and the major complication rate was 2%. However, these lower rates can be attributed to the fact that all procedures are performed by the same surgeon. Experience gained by the first generation of surgeons has helped newer surgeons to minimize complications and shorten operative time [25–27] and has presumably led to better oncologic results with improved preservation of quality of life [28, 29].

In the present study, as the surgeon progressed with his experience multiple modifications were done with his technique. Although it is thought to be quite meaningful to investigate the effects of these modifications, it seems to be difficult to compare the results of each modification because of our small cohort.

In conclusion, the results of this large series of 100 patients are promising. The advantages of minimally invasive surgery and radical retropubic prostatectomy seem to be combined in extraperitoneal LRP. In addition, the recently introduced intrafascial nerve-sparing technique further improves outcomes for the procedure.

## 5. Conclusions

In the present series, results from 100 LRPs demonstrated satisfactory results in terms of complications, urinary continence, or oncologic outcomes. Long-term followup is still needed to address the oncologic effectiveness of the laparoscopic approach.

## Figures and Tables

**Table 1:** Overall pretreatment characteristics of all 100 patients.

Variable	Mean ± SD
Age (years)	65.2 ± 4.9
BMI (kg/m^2^)	23.3 ± 2.4
PSA (ng/mL)	7.6 ± 2.8

*Clinical stage*	*n*
T1c	93
T2a	7
Preoperative hormonal therapy	4

**Table 2:** Surgical factors for all 100 patients and corresponding percentage of positive margin rates.

Variable	*n*	Positive margins
Pathological Gleason score		
≤6	12	1 (8.3%)
7	77	25 (32.5%)
8–10	11	6 (54.5%)

Pathological stage		
pT0	1	0 (0.0%)
pT2a	8	0 (0.0%)
pT2b	64	15 (23.4%)
pT3a	24	17 (70.8%)
pT3b	3	0 (0.0%)

**Table 3:** Complications related to the surgical experience.

Complications	Cases				Total
	1–25	26–50	51–75	76–100	
Intraoperative					
Rectal injury		1			1

Immediate postoperative					
Urine collected by drain	1				1

Long-term complications					
Inguinal hernia	3	6	2	1	12
Urethral stricture (fossa navicularis)		1	1		2
Vesicourethral anastomotic stricture			1	1	2
